# A Bacterial Surface Display System Expressing Cleavable Capsid Proteins of Human Norovirus: A Novel System to Discover Candidate Receptors

**DOI:** 10.3389/fmicb.2017.02405

**Published:** 2017-12-06

**Authors:** Qian Xu, Pei’en Ni, Danlei Liu, Yujie Yin, Qianqian Li, Jvmei Zhang, Qingping Wu, Peng Tian, Xianming Shi, Dapeng Wang

**Affiliations:** ^1^MOST-USDA Joint Research Center for Food Safety, School of Agriculture and Biology, Shanghai Jiao Tong University, Shanghai, China; ^2^Department of Bioengineering, Shanghai Institute of Technology, Shanghai, China; ^3^State Key Laboratory of Applied Microbiology Southern China, Guangdong Provincial Key Laboratory of Microbial Culture Collection and Application, Guangdong Open Laboratory of Applied Microbiology, Guangdong Institute of Microbiology, Guangzhou, China; ^4^Produce Safety and Microbiology Research Unit, Western Regional Research Center, Agricultural Research Service – United States Department of Agriculture, Albany, CA, United States

**Keywords:** human noroviruses, GII.4, cell surface display, P proteins, histo-blood group antigens, receptor

## Abstract

Human noroviruses (HuNoVs) are the dominant cause of food-borne outbreaks of acute gastroenteritis. However, fundamental researches on HuNoVs, such as identification of viral receptors have been limited by the currently immature system to culture HuNoVs and the lack of efficient small animal models. Previously, we demonstrated that the recombinant protruding domain (P domain) of HuNoVs capsid proteins were successfully anchored on the surface of *Escherichia coli* BL21 cells after the bacteria were transformed with a plasmid expressing HuNoVs P protein fused with bacterial transmembrane anchor protein. The cell-surface-displayed P proteins could specifically recognize and bind to histo-blood group antigens (HBGAs, receptors of HuNoVs). In this study, an upgraded bacterial surface displayed system was developed as a new platform to discover candidate receptors of HuNoVs. A thrombin-susceptible “linker” sequence was added between the sequences of bacterial transmembrane anchor protein and P domain of HuNoV (GII.4) capsid protein in a plasmid that displays the functional P proteins on the surface of bacteria. In this new system, the surface-displayed HuNoV P proteins could be released by thrombin treatment. The released P proteins self-assembled into small particles, which were visualized by electron microscopy. The bacteria with the surface-displayed P proteins were incubated with pig stomach mucin which contained HBGAs. The bacteria-HuNoV P proteins-HBGAs complex could be collected by low speed centrifugation. The HuNoV P proteins-HBGAs complex was then separated from the recombinant bacterial surface by thrombin treatment. The released viral receptor was confirmed by using the monoclonal antibody against type A HBGA. It demonstrated that the new system was able to capture and easily isolate receptors of HuNoVs. This new strategy provides an alternative, easier approach for isolating unknown receptors/ligands of HuNoVs from different samples including mammalian cell lines, oysters, and fresh produce.

## Introduction

Noroviruses (NoVs) are non-enveloped, single-stranded, positive-sense RNA viruses in the Caliciviridae family (Jiang et al., 1993). NoVs have been sub-divided into seven genogroups (GI-GVII), based on the genomic sequence of its major capsid protein (VP1). NoV GI, GII, and GIV are capable of infecting humans, comprising the human noroviruses (HuNoVs) (Vinje, 2015). HuNoVs are the main cause of human non-bacterial gastroenteritis worldwide (Hoa Tran et al., 2013). In the United States, it is estimated that 59% foodborne illnesses were caused by HuNoVs each year (Scallan et al., 2011). Of the confirmed norovirus outbreaks, 86% cases were caused by HuNoV GII strains during 2009–2012 in United States (Hall et al., 2014).

Fundamental research on HuNoVs has been long-hampered by the inability to efficiently culture the viruses *in vitro*. Despite recent developments that have allowed HuNoVs to be replicated in human B-cells (Jones et al., 2015) and stem-cell-derived human enteroids (Ettayebi et al., 2016), *in vitro* culturing of HuNoV remains too immature for general applications. Instead, Tulane virus (TV), feline calicivirus (FCV), and murine norovirus (MNV) have often been utilized as surrogates for HuNoVs (Hirneisen and Kniel, 2013; Wang et al., 2014; Farkas, 2015). Recombinantly expressed HuNoVs capsids, also known as virus-like particles (VLPs), are morphologically and antigenically similar to the viruses have also been utilized for the study of viral immunogenicity and host–receptor interactions (Gray et al., 1993; Green et al., 1993; Hutson et al., 2003; Huang et al., 2005). While insect cell culture-expressed ORF2 protein spontaneously form empty VLPs with morphological and antigenic similarities to viral particles (Green et al., 1993; Prasad et al., 1999), the overall process of producing recombinant baculoviruses for use in eukaryotic expression systems remains difficult and time-consuming (Jiang et al., 1992). Meanwhile, expression of the protruding domain (P domain) of ORF2 in prokaryotic system could produce P proteins that self-assemble into P particles. The P particles are made of 12 dimers of the expressed P domains (Tan et al., 2008). Saliva-based receptor binding assay showed that P particles retain binding capability to human histo-blood group antigens (HBGAs), which have been considered as receptor/co-receptor for HuNoVs (Huang et al., 2003, 2005; Hutson et al., 2004; Tan and Jiang, 2005a). The HBGAs binding affinity of P particles is comparable to that of VLPs, and is much stronger than that of P dimers (Tan and Jiang, 2005b; Tamminen et al., 2012). In addition, P particles are excellent platforms for the study of antigen presentation (Tan et al., 2011; Tan and Jiang, 2012). Unfortunately, both VLPs and P particles are unusable for the isolation of the virus-ligand/receptor complex (Tan and Jiang, 2005b; Su et al., 2015).

**FIGURE 1:**
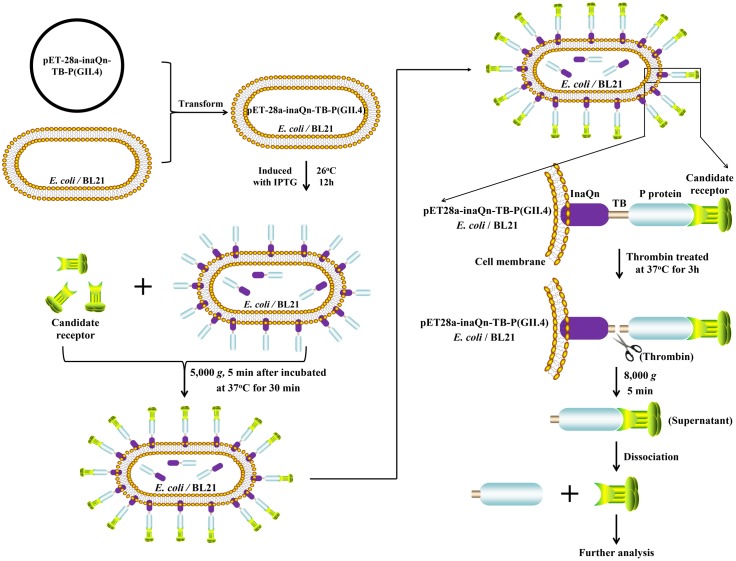
Schematic overview of the novel system for isolating bacterial–viral receptor-complex. 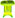: Candidate receptor; 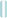: P protein (GII.4); 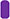: InaQn protein; 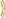: cell membrane of *Escherichia coli* (BL21).

We have previously reported that HuNoV VP1 and P proteins can be displayed on the surface of *Escherichia coli* by appending its sequence to the N-terminal domain sequence of bacterial ice-nucleation protein (INP) (Niu et al., 2015). Bacterial INP is member of a family of proteins that allows Gram-negative bacteria to promote ice crystal formation at relatively high temperatures (Kawahara, 2002), and is comprised of three distinct structural domains: N-terminal domain, highly-repetitive central domain, and C-terminal domain. It has been reported that INP’s N-terminal domain (InaQn) is responsible for the transmembrane transport and outer-membrane-binding activity (Shimazu et al., 2003; Li et al., 2012). Our early studies show that bacterial-surface-displayed P proteins retains the ability to recognize and bind HBGAs (Niu et al., 2015). However, this bacterial-surface-P-protein-display-system could not be directly used for the analysis of candidate receptors, as the biochemical complexity of the present-and-attached bacteria would completely overwhelm any attempts at characterization of the isolated viral receptors. In this study, we have surmounted the fore-mentioned hurdle to applying the bacterial-surface-P-protein-display system in a viral receptor isolation context by adding a thrombin-susceptible domain to the existing construct to facilitate release of the P-protein-candidate-receptor complex from bacteria to enable easy purification (**Figure 1**).

## Materials and Methods

### Bacterial Strains and Construction of the Recombinant Plasmids

Competent *E. coli* DH5α and BL21 (Thermo Fisher, Shanghai, China) were used for recombinant plasmid construction and protein expression, respectively. Oligonucleotide “TB” coding for the peptide sequence of Leu-Val-Pro-Arg-Gly-Ser, was synthesized by Suzhou GENEWIZ Bio-Technology, Co., Ltd. Then, the artificially synthesized *inaQn-TB* sequence was inserted into cloning vector pUC57 to create pUC57-inaQn-TB. After re-digestion of plasmids pET28a, pET28a-P (GII.4) and pUC57-inaQn-TB (**Table 1**), the *inaQn*-*TB* and *P* (GII.4) fragment were inserted into *pET-28a* to create recombinant plasmid pET28a-inaQn-TB-P (GII.4). Similarly, pET28a-inaQn-TB was constructed from pET28a-inaQn for use as a negative control. All the recombinant plasmids were used to transform bacteria *E. coli* BL21.

**Table 1:** Recombinant plasmids with restriction enzyme sites.

Recombinant plasmids	Restriction enzyme sites	The length of nucleic acid fragments (bp)
pET28a	*Nco* I and *Eco*R I	5265
pUC57-inaQn-TB	*Nco* I and *Bgl* II	893 (inaQn-TB)
pET28-P (GII.4)	*Bgl* II and *Eco*R I	948 (GII.4 P protein)

### Culture and Expression of InaQn-TB-P (GII.4) Fusion Proteins in *E. coli* BL21

*Escherichia coli* BL21 was transformed with recombinant plasmids pET28a-inaQn-TB-P (GII.4) or pET28a-inaQn-TB. The recombinant bacteria were each cultured in Luria-Bertani (LB) (0.5% yeast extract, 1.0% trypton, and 1.0% NaCl) liquid medium containing 100 μg/mL kanamycin, at 37°C with shaking (150 rpm) for overnight. One hundred microliters (100 μL) of each overnight culture were added to aliquots of new LB medium (10 mL, w/100 μg/mL kanamycin), and were cultured at 37°C with shaking (150 rpm) until OD_600_ reached ∼0.6. Isopropyl β-D-1-thiogalactopyranoside (IPTG; Merck, Germany) was added to each OD_600_ ∼0.6 culture to a total concentration of 0.5 mM, and incubated at 26°C with shaking (120 rpm) for 12–16 h. The induced-and-expressed cultures were stored at 4°C for further use.

### Releasing Soluble P Proteins by Thrombin Digestion

Recombinant *E. coli* BL21 expressing inaQn-TB-P in culture (as described above) was adjusted to an OD_600_ of 1.0. The bacteria were isolated and washed twice with phosphate-buffered saline (PBS), then resuspended in digestion buffer (1.0 mL, 20 mM Tris-HCl and 150 mM NaCl, pH 8.0). In accordance with manufacturer guidelines for enzymatic activity, Bovine thrombin (Yeason, Shanghai, China) was added at 1: 2,000 (e.g., 2.0 U enzyme was added to 1.0 mg target protein) to each digestion reaction and incubated at 37°C for 3 h. The reaction was centrifuged at 4°C at 8,000 RCF for 5 min, and the supernatant was quantified for P protein by a commercial BCA assay kit (Beyotime, Shanghai, China). The protein was stored at -20°C for further use.

### SDS–PAGE and Western Blot

Recombinant *E. coli* BL21 strains containing plasmid constructs pET28a-inaQn-TB-P, pET28a-inaQn-P, and pET-28a (negative control) were induced with IPTG, washed twice and then resuspended in PBS. Surface-expressed P proteins from induced BL21-pET28a-inaQn-TB-P (GII.4) were released from bacteria by thrombin digestion as described in the previous section. For SDS-PAGE, the IPTG-induced bacteria were dissolved in 2× SDS-PAGE loading buffer (100 mM, pH 6.8 Tris-HCl, 4% SDS, 20% Glycerol, 0.2% Bromophenol Blue, 2% DTT), while the thrombin-released P proteins were dissolved in 5× SDS-PAGE loading buffer (250 mM, pH 6.8 Tris-HCl, 10% SDS, 50% Glycerol, 0.5% Bromophenol Blue, 5% DTT). Each sample was boiled for 5 min, and 10.0 μL from each was loaded and separated in a 12% SDS-PAGE gel, followed by staining with Coomassie Blue R250 (Beyotime, Shanghai, China). Western Blotting was conducted as described in a previous report (Wang et al., 2008). The antibody against GII.4 HuNoV recombinant viral capsid protein (1: 5,000; Niu et al., 2015), and peroxidase-conjugated goat anti-mouse IgG (H+L, 1: 3,000; Yeasen, Shanghai, China) were used as primary and secondary antibodies in Western Blotting as described in our previous publication (Niu et al., 2015). The 3, 3′-diaminobenzidine (Fedbio, Wuhan, China) was used as chromogenic substrate.

### Visualization of P Proteins by Electron Microscopy (EM)

Thrombin-released P proteins were visualized by EM as previously described by Tan and Jiang (2005b) with minor modifications. Thrombin-released P protein was quantitated by BCA assay kit (Beyotime, Shanghai, China). Twenty microliters of thrombin-released P protein (200 μg/mL) was loaded onto the support grid (Mainstream, Shanghai, China) and allowed to bind for 2 min. The remaining solution was wicked away with wedges of Whatman filter paper (General Electric, Beijing, China). Twenty microliters of 1.0% phosphotungstic acid (Ted Pella, Redding, CA, United States) was added to the support grid and allowed to stain for 2 min. The remaining stain was wicked away with wedges of Whatman filter paper. The support grid was washed with 20 μL of ddH_2_O and dried immediately by wicking with Whatman filter paper. The support grid was examined under an H-7650 microscope (Hitachi, Japan) at 50,000× magnification and 80 kV.

### Measuring the HBGA-Binding Ability of Released P Proteins

Thrombin-released P protein was serially-diluted in digestion buffer to a series spanning 2 to 200 μg/mL. One hundred microliters of each dilution were added into immunoassay wells (Nunc Immuno Module, VWR, San Francisco, CA, United States) and incubated at 4°C for overnight. The coated wells were washed three times with PBS, then blocked with 1.0% bovine serum albumin (“BSA”; Yeason, Shanghai, China) at 37°C for 1 h. The blocked wells were washed three times with 120.0 μL of PBS-T (PBS containing 0.1% Tween-20, pH 7.2). One hundred microliters of 1.0 mg/mL Type III porcine gastric mucin (“PGM”; Sigma, St. Louis, MI, United States) was added to each well and incubated at 37°C for 30 min. The optimal dilutions of anti-type A HBGA monoclonal antibody BG2 (Covance, Emeryville, CA, United States) and the secondary antibody, a peroxidase-conjugated goat anti-mouse IgG (H+L chains; Yeasen, Shanghai, China) were determined experimentally to be 1: 1,000 and 1: 3,000 in blocking buffer, respectively. Both antibodies were incubated at 37°C for 1 h respectively. In addition, the antibody against the recombinant viral capsid proteins from our previous report was also utilized as a primary antibody to detect P proteins (data not shown) (Niu et al., 2015). Wells were washed four times with PBS-T following each step. After incubation with 100.0 μL of 3,3′,5,5′-tetramethylbenzidine (TMB; Fedbio, Wuhan, China) in the dark for 10 min, the chromogenic reaction was halted using 50.0 μL of 2 mol/L H_2_SO_4_. The OD_450_ values were measured using a Sunrise Microplate Reader (Tecan Sunrise, Switzerland). Cells transformed with recombinant plasmid pET28a-inaQn-TB were treated in the same way and used as negative controls. Samples were considered positive when the positive to negative (P/N) ratio was greater than 2.0.

### Identification of Type A HBGA Captured by Surface-Displayed HuNoV P Proteins by Enzyme-Linked Immunosorbent Assays (ELISA)

IPTG-induced BL21 with recombinant plasmid pET28a-inaQn-TB-P (GII.4) were collected by centrifugation at 5,000 RCF for 5 min. The pellet was washed twice with PBS (pH 7.2) and adjusted to an OD_600_ of 1.0. The washed cultures were incubated at 37°C for 30 min with PGM at final concentrations of 0.1, 0.2, 0.5, and 1.0 mg/mL. The solution of putative bacteria-PGM complex was washed with PBS containing 0.5‱ Tween-20 (pH 7.2) at least three times to remove any un-complexed PGM. The putative bacteria-PGM complex was digested by bovine thrombin (100 U/mL, Yeason, Shanghai, China) at 37°C for 3 h as described above. The supernatant containing the thrombin-released PGM-to-P-protein complex was collected by centrifugation at 4°C at 8,000 RCF for 5 min. Each immunoassay well (Nunc Immuno Module; VWR, San Francisco, CA, United States) was incubated with 100.0 μL of the supernatant at 4°C for overnight. Each well was washed three times with PBS (pH 7.2), blocked with 120.0 μL of 1.0% BSA in PBS at 37°C for 1 h, and then washed three times with PBS again. One hundred microliters of anti-type A HBGA monoclonal antibody BG2 (1: 1,000; Covance, Emeryville, CA, United States) was added to each well. Peroxidase-conjugated goat anti-mouse IgG (H+L chains, 1: 3,000; Yeasen, Shanghai, China) was used as the secondary antibody. All antibody incubation steps were performed at 37°C for 1 h. The wells were washed three times with 120.0 μL of PBS-T after each incubation step. Then, 100.0 μL of TMB (Fedbio, Wuhan, China) was added to each well. After incubating in the dark for 10 min, the chromogenic reaction was halted using 50.0 μL of 2 mol/L H_2_SO_4_, and the OD_450_ values were measured. Bacteria with recombinant plasmids pET28a-inaQn-TB and pET-28a were also tested in the same way. Cells incubated without PGM were used as negative controls.

### Statistical Analysis

IBM SPSS statistics software (version 19) was used for statistical analysis. Each experiment was performed in triplicate (*N* = 3), which in turn was independently repeated three times (*n* = 3). One-way ANOVA was utilized for data analysis. Differences in means were considered significant when *p* < 0.05.

## Results

### Characterization of Thrombin-Released HuNoV P Proteins

**FIGURE 2:**
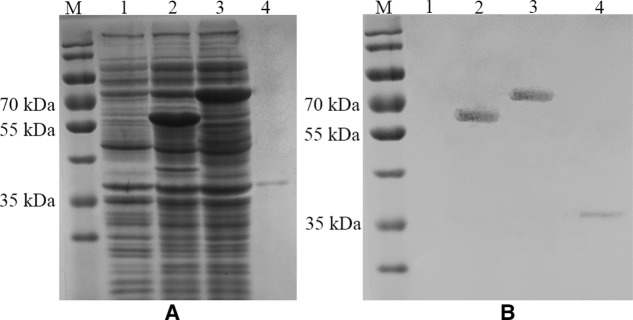
SDS-PAGE **(A)** and Western Blot **(B)** analysis of the expression of *E. coli* cell surface display systems for HuNoV (GII.4) recombinant capsid proteins with (3) or without the linker fragment (2) and the thrombin-released P proteins (4). M: prestained protein ladder (Catalog No.: 26616, Thermo Fisher, Shanghai, China); (1) pET-28a/BL21; (2) pET28a-inaQn-P (GII.4)/BL21; (3) pET28a-inaQn-TB-P (GII.4) /BL21; (4) Thrombin-released P proteins.

SDS-PAGE and Western Blot were used to characterize the expression of the fusion proteins and the thrombin-released P protein. The thrombin-released P protein, InaQn-P and InaQn-TB-P fusion proteins were expected to be segregated to positions roughly corresponding to 35, 58, and 70 kDa, respectively. All proteins were visualized by Coomassie Blue staining in SDS-PAGE (**Figure 2A**), and confirmed by Western Blot (**Figure 2B**). We found that the thrombin-released P proteins did not exhibit a significant background of bacterial proteins (**Figure 2A**).

### The Morphology of Released HuNoV P Proteins under EM

**FIGURE 3:**
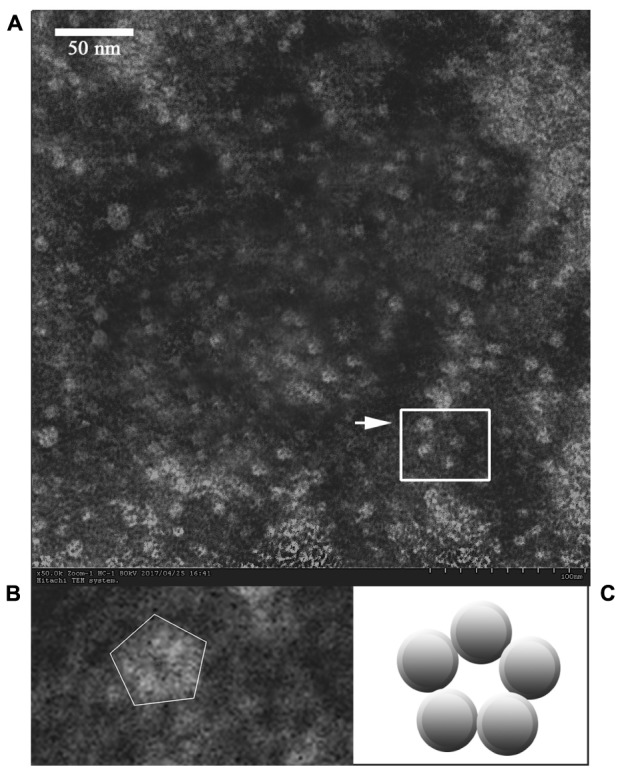
Electron micrograph of the released P proteins. **(B)** Is the enlargement of the center part of **(A)**, and the arrow indicates a pentagonal-ring structure for the particles. **(C)** Is a model of the cross-section of the P particle.

The morphology of the thrombin-released P proteins was observed by negative-staining EM. The P protein was revealed to have a spherical structure of ∼15 nm (**Figure 3**).

### The Thrombin-Released P Proteins Could Bind to HBGA

**FIGURE 4:**
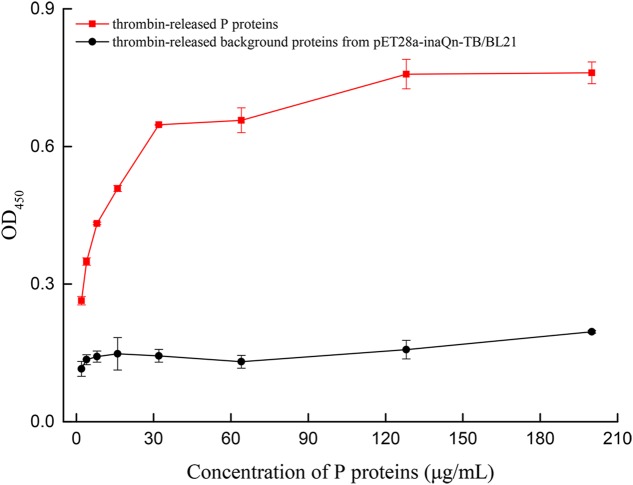
OD of type A HBGA bound to serially diluted thrombin-released P proteins (red) and the thrombin-released background proteins from pET28a-inaQn-TB/BL21 (black). Bars represent standard errors.

A modified ELISA was developed to quantitate the type-A HBGA-binding capacity of thrombin-released P proteins. The binding capacity was presented by OD readings (**Supplementary Table S1**) and P/N ratios. A positive correlation was observed between OD_450_ and the concentration of thrombin-released P proteins, until a plateau was reached at 128 μg/mL of the latter (**Figure 4**). The OD_450_ P/N ratios of P-protein-bound-HBGAs vs. pET28a-inaQn-TB/BL21 background proteins were: 2.29, 2.58, 3.04, 3.43, 4.50, 5.02, 4.82, and 3.88 for thrombin-released P proteins at concentration of 2, 4, 8, 16, 32, 64, 128, and 200 μg/mL, respectively. The optimal concentration of P protein to bind HBGAs was 64 μg/mL. There was no significant binding of HBGA to the thrombin-digested expression product from pET28a-inaQn-TB/BL21, which does not express any HuNoV P protein domains (**Figure 4**). As shown in **Figure 4**, the P proteins could be directly collected by low speed centrifugation after thrombin treatment, and maintain its ability to bind to its receptors.

### Isolation and Characterization of Receptors from HBGAs-HuNoV P Proteins Complex

**FIGURE 5:**
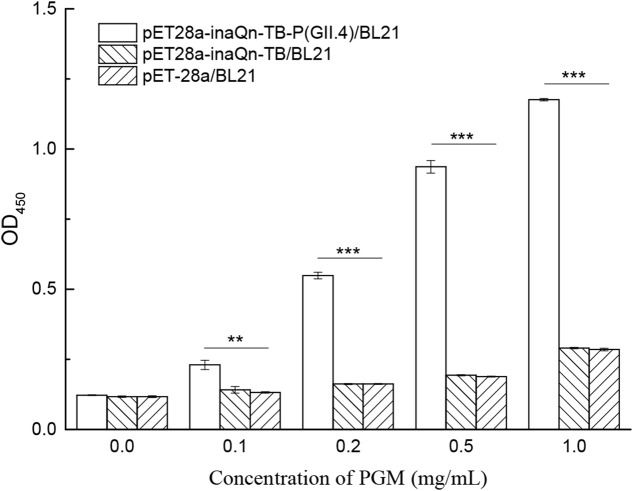
Detection of type A HBGA released from the HuNoV P protein–receptor complex incubated with various concentrations of PGM. ^∗∗^0.01 < *p <* 0.05; ^∗∗∗^*p <* 0.01; Bars represent standard errors.

Co-incubation of viral receptor (HBGA) and induced recombinant bacteria resulted in the binding of HBGA to surface-displayed HuNoV P proteins. The bacteria-HuNoV P proteins-HBGAs complex could be isolated by a low-speed centrifugation step (5,000 RCF for 5 min). The HuNoV P protein-HBGA complex can be released and isolated from bacteria by thrombin-digestion, followed by low speed centrifugation (8000 RCF, 5 min), as indicated in **Figure 1**. A modified ELISA was developed to detect type A HBGA isolated from the complex (**Figure 5**). The amount of the viral receptor in the complex was reflected by OD_450_ readings with MAb against type A HBGA (BG2) (**Supplementary Table S2**). The P/N ratio of recombinant cells incubated with or without PGM was calculated. Various concentrations of PGM were used to test for an optimal concentration to produce the lowest receptor background for the HBGAs-HuNoV-P proteins–bacteria complex. Although there was a positive correlation between OD_450_ and PGM concentration, OD_450_ of the background increased significantly when concentration of the latter exceeded 0.5 mg/mL. Incubation of PGM concentrations of 0.1, 0.2, 0.5, and 1.0 mg/mL with pET28a-inaQn-TB-P (GII.4)/BL21 produced P/N ratios of 1.88, 4.49, 7.66, and 9.61, respectively; similarly, pET28a-inaQn-TB/BL21 produced P/N ratios of 1.20, 1.38, 1.65, and 2.48, respectively; and that of pET-28a/BL21 produced P/N ratios of 1.13, 1.39, 1.61, and 2.44, respectively. These results indicate that the optimum concentration of PGM for the binding test was about 0.5 mg/mL. The absorbance of type A HBGA recovered from the complex was significantly higher than that of pET28a-inaQn-TB/BL21 and pET-28a/BL21 (*p* < 0.05). There was no significant difference between signals of *E. coli* BL21 cells transformed with plasmid pET28a-inaQn-TB and that of plasmid pET-28a with or without PGM treatment (*p* > 0.05). These results indicated that InaQn-TB-P protein could be displayed on the surface of bacterial cells, retained its receptor-binding ability, and that the receptor-to-P protein complex could be easily isolated from the system.

## Discussion

Foodborne illness is a persistent problem worldwide that exposes the public to the risk of infection and causes economic losses (Orchard et al., 2016). The majority of gastroenteritis outbreaks are caused by HuNoV GII.4 strains (Scallan et al., 2011). HuNoV outbreaks are often associated with oysters (Wang et al., 2008; Berger et al., 2010; Ma et al., 2017), strawberries (Le Guyader et al., 2004; Sarvikivi et al., 2012) and romaine lettuce (Ethelberg et al., 2010; Esseili et al., 2012). The identification and analysis of viral receptors are the key to exploring the mechanism of virus invasion and host infection. Recently, CD300If and CD300Id were identified as functional receptors of MNV (Orchard et al., 2016). Zhang et al. (2015) reported TV recognized the Type A3 and B HBGA. Tan et al. (2015) reported that sialic acids could be used as additional cellular receptors/co-receptor of Tulane virus and HuNoVs. It is known that receptor-like molecules for HuNoVs are present on the surface of produce, oyster gastrointestinal cells and blood cells (Tian et al., 2006; Gao et al., 2016; Wang et al., 2017), and might facilitate the bioaccumulation of HuNoVs (Tian et al., 2006; Wang et al., 2012). HuNoV’s adherence to receptor-like molecules is resistant to washing, which may facilitate its bioaccumulation on produce to reach potentially human-infectious doses despite low-concentration contamination sources (Wang et al., 2012; Zhou et al., 2017). Unfortunately, the mechanisms of interaction between viruses and fresh produce are still poorly understood.

It is difficult for most labs to collect HuNoVs in sufficient quantity to perform direct studies. Instead, VLPs and P particles have been widely-used for studies of viral antigenicity, viral immunogenicity and interactions between viral particles and its receptors (Tan et al., 2004, 2011). However, VLPs and P particles have limited applications toward the isolation of viral candidate receptors due to the fact that these water-soluble viral particle-ligand complexes cannot be isolated using simple techniques, such as low-speed centrifugation (Jiang et al., 1992).

Previously, an expression system had been constructed utilizing InaQn to display recombinant HuNoV capsid proteins on the bacterial surface (Niu et al., 2015). With the help of the InaQn, the target protein could be directly displayed on the surface of the bacteria transformed by a recombinant plasmid encoding the fusion target protein (Kim and Yoo, 1999; Kwak et al., 1999; Cochet and Widehem, 2000; Li et al., 2009). In our previous study, we demonstrated that bacterial surface-displayed P proteins could specifically recognize and bind HBGAs (Niu et al., 2015). The bacterial surface display system could interact with HBGA-like molecules from romaine lettuce (Wang et al., 2017). However, the fact that the bacterial surface-displayed P proteins remain anchored to *E. coli* greatly complicates any further purification and analysis of receptor candidates. In this study, the existing expression system was modified by adding a thrombin-cleavable linker fragment between the membrane-embedded carrier protein (InaQn) and the surface-displayed P protein (**Figure 1**).

We have previously confirmed the presence of type A and H1 HBGAs in PGM (Tian et al., 2007). PGM has been used to capture HuNoVs, and for evaluating the binding efficiency between viral receptors and surface-displayed P proteins (Tan and Jiang, 2005b). The results of the present study indicate that the bacterial surface-displayed P proteins could bind to HBGAs, and this binding was due to specific recognition (**Figure 5**).

The present study demonstrates that the modified and improved surface display system could be used to purify P proteins (**Figures 1**, **2B**, **3A**). Unlike other prokaryotic systems used to express P particles (Jiang et al., 1992; Tan and Jiang, 2005b), the expressed and displayed P proteins of the thrombin-cleavable-linker modified system could be easily released from the bacterial surface by thrombin treatment (**Figure 2B**). The thrombin-released putative P proteins were confirmed by ELISA, SDS-PAGE, Western Blot, and visualized by EM (**Figures 2**–**4**). We were able to show by EM that the thrombin-released P proteins could spontaneously assemble into particles with ring or pentagonal-shaped structures (**Figure 3**), which is similar to that of prior reports characterizing the behavior of recombinant P proteins expressed and purified using a much more time-consuming protocol (Tan and Jiang, 2005b). Our thrombin-released P proteins could be easily collected by centrifugation, without complex purification steps, saving significant time and labor. This general strategy may provide for a new approach to express and purify proteins in the future.

This study shows that adding a thrombin-susceptible domain to an existing surface-expression fusion construct maintains surface expression, and allows the surface-expressed domain to be released from the bacteria by thrombin treatment. The novel system was used to isolate HBGAs (**Figure 5**), a known receptor of HuNoVs. The bacterial-surface-displayed-P-protein-to-HBGAs complex can be concentrated and clarified with only low-speed centrifugation. The P-protein-to-HBGAs complex can be released and isolated from bacteria by thrombin treatment, followed by low-speed centrifugation. This can facilitate analysis of candidate receptors free from the biochemical background of *E. coli*. We observed that the thrombin-released complex could be recognized by both MAb (BG2) for HBGA and polyclonal antibodies (Niu et al., 2015) against P proteins (data not shown). This indicates that the HBGAs bound to viral proteins were stable after thrombin digestion and could be recognized by MAb. Currently, we are in the process of constructing a cleavable P protein with a polyhistidine-tag, which will make concentration and purification of the thrombin-released complex more efficient. We are also in the process of isolating viral receptors from cell lines that support replication of TV and HuNoVs. In our current studies, this improved system for isolating candidate receptors is used to discover and analyze the specific ligands/receptors in lettuce leaf, oyster tissues, or cell lines. For example, when candidate receptors of HuNoV in oysters were mixed with P-protein-surface-displayed bacteria, receptor-P-protein-bacteria complex was formed. The soluble contents from oyster could be removed by a low speed centrifugation step. After receptor-P-protein complex was released by enzyme, they were easily separated from insoluble contents from oyster and bacteria by another round of low speed centrifugation. The advanced bacterial cell surface display system can be applied to not only HuNoVs, but also to Rotavirus and other yet uncultivable (*in vitro*) viruses in the future. We believe this advanced system provides a novel approach to discover unknown receptors or capsid-binding ligands for HuNoVs as well as other pathogens.

## Author Contributions

DW, QL, and PT designed the experiments. QX, PN, DL, YY, and DW carried out experiments. JZ, QW, DW, and PT analyzed sequencing data and experimental results. QX, QL, DW, and PT wrote and modified the manuscript. XS provided laboratory equipment and place.

## Conflict of Interest Statement

The authors declare that the research was conducted in the absence of any commercial or financial relationships that could be construed as a potential conflict of interest.

## Supplementary Material

TABLE S1 The OD reading data of type A HBGA bound to P proteins and background proteins.

TABLE S2 The OD reading data of type A HBGA isolated from thrombin- released complex.
